# The complete mitochondrial genome of an Atlantic Ocean Shortfin Mako Shark, *Isurus oxyrinchus*

**DOI:** 10.1080/23802359.2019.1677524

**Published:** 2019-10-18

**Authors:** Jonathan Gorman, Nicholas Marra, Mahmood S. Shivji, Michael J. Stanhope

**Affiliations:** a Department of Population Medicine and Diagnostic Sciences, College of Veterinary Medicine, Cornell University, Ithaca, NY, USA;; b Department of Biology, Drury University, Springfield, MO, USA;; c Save Our Seas Shark Research Center and Guy Harvey Research Institute, Nova Southeastern University, Dania Beach, FL, USA

**Keywords:** *Isurus oxyrinchus*, mitochondrial genome, shortfin mako

## Abstract

We report the first complete mitochondrial genome of a shortfin mako shark from the Atlantic Ocean. The genome had 16,700 base pairs and contained 13 protein-coding genes, 2 rRNA genes, 22 tRNA genes, and a non-coding D-loop. There were 81 individual differences compared to the published mitochondrial genome of a shortfin mako from the Pacific Ocean, with most variability found in protein coding genes, especially ND5, ND3, and ND1. These highly variable genes may be useful population markers in future studies, and availability of a second mitogenome will assist with future, genome-scale studies of this IUCN Endangered species.

Shortfin mako sharks are globally distributed in pelagic, temperate and tropical waters, and this species is listed as Endangered by the IUCN (Rigby et al. 2019). Limited mitochondrial studies of shortfin makos have indicated matrilineal structuring across large geographic scales (Heist et al. 1996; Corrigan et al. 2018), but microsatellite studies have produced mixed results regarding the connectivity between Atlantic and Pacific populations (Schrey and Heist 2003; Corrigan et al. 2018). The only published shortfin mako mitochondrial genome (mitogenome) available is a Pacific specimen (Chang et al. 2013). The addition of the first Atlantic-sourced mitogenome will aid future studies of this endangered species based on genome-scale data.

We acquired heart tissue from an adult male shark captured in 2013 off the coast of Florida (geospatial coordinates: 30.0625, −76.9194), and the tissue is stored under accession number OC-298 at Nova Southeastern University, College of Natural Sciences and Oceanography. We extracted DNA from this tissue and sequenced a genomic library using two lanes of 250 bp PE on an Illumina HiSeq2500. Mapping assembly was conducted using MITObim v.1.9 (Hahn et al. 2013) with the Pacific specimen mitogenome as a reference. We annotated the assembly using DOGMA (Wyman et al. 2004), with annotations verified through comparisons to mitogenomes of other Lamnidae sharks (Figure 1). This mitogenome is deposited as MF537044 (BioSample SAMN04526263).

The Atlantic shortfin mako mitogenome was 16,700 bp and had the following composition: 28.8% A, 28.0% C, 15.2% G, and 28.0% T with a GC content of 43.2%. It contained 2 rRNA genes, 22 tRNA genes, 13 protein-coding genes, and a non-coding region. The Atlantic and Pacific mitogenomes (RefSeq: NC_022691.1) have a sequence identity of 99.5% with 81 polymorphisms. Three of these polymorphisms were in rRNA genes, 3 were in tRNA genes, 9 were in non-coding regions (8 in D-loop), and 66 were in protein-coding genes. All polymorphisms were SNPs except for one indel in the 12S rRNA region. Only 10 of the polymorphisms in protein-coding genes were nonsynonymous. Some of the individual genes had a larger number of Pacific vs. Atlantic differences, including ND5, ND3, and ND1 with sequence identities of 99.07%, 99.20%, and 99.28% respectively, which are all lower than the sequence identity across the entire mitogenomes. The genetic differences between the Atlantic and Pacific mitogenomes may be potential markers for distinguishing the two populations in future studies.

We also analysed genetic differences between two published mitochondrial genomes (RefSeq: NC_022415.1 and gb: KX389266.1) from the con-familial white shark (IUCN Vulnerable) from the Pacific and Atlantic to see if the protein-coding genes that were the most variable in shortfin makos were also the most variable in white sharks. The 13 protein-coding genes were ranked by sequence identity for both species and a Friedman test returned a marginally significant *p*-value of 0.0857. This indicates that there may be consistency amongst lamnid sharks in terms of which mitrochondrial genes have the most variation, although more comparisons are needed to confirm this finding. If these genes are consistently variable, they might be useful as genetic markers in other lamnid shark species, some of which are fishery exploited.

## Figures and Tables

**Figure 1.**
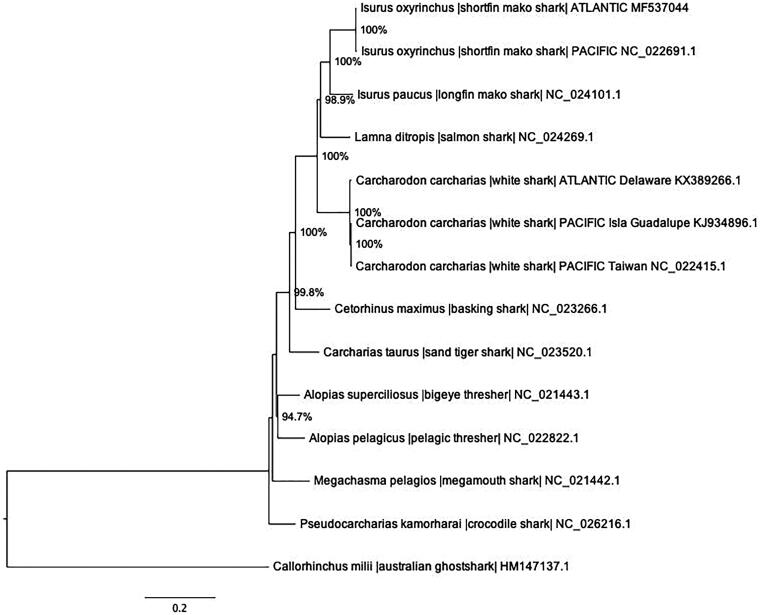
We used MEGA7 (Kumar et al. 2016) to align the mitochondrial genomes of 11 species of chondrichthians (10 Lamniformes), including 3 individual white sharks and 2 individual shortfin mako sharks. The alignment was used to produce a maximum likelihood tree based on the GTR model of evolution and 1000 bootstraps. The tree with the highest log likelihood is shown. The percentage of trees in which the associated taxa clustered together is shown next to the branches for all nodes where the support is >75%. The tree is drawn to scale, with branch lengths measured in the number of substitutions per site. Our shortfin mako sequence clustered with the Pacific shortfin mako sequence. The root of the tree was placed between the Australian ghostshark, representing the outgroup, and the elasmobranchs.
